# Composite Flour from Indonesian Local Food Resources to Develop Cereal/Tuber Nut/Bean-Based Ready-to-Use Supplementary Foods for Prevention and Rehabilitation of Moderate Acute Malnutrition in Children

**DOI:** 10.3390/foods10123013

**Published:** 2021-12-05

**Authors:** Fetriyuna Fetriyuna, Ratna Chrismiari Purwestri, May Susandy, Realm Köhler, Ignasius Radix A. P. Jati, Nia Novita Wirawan, Hans-Konrad Biesalski

**Affiliations:** 1 Institute of Nutritional Science (140a), University of Hohenheim, Garbenstrasse 30, 70599 Stuttgart, Germany; Alis.R@uni-hohenheim.de (R.K.); biesal@uni-hohenheim.de (H.-K.B.); purwestri@fld.czu.cz (R.C.P.); 2 Department of Food Technology, Faculty of Agro-Industrial Technology, Universitas Padjadjaran, Jln. Raya Bandung-Sumedang Km. 21, Jatinangor, Sumedang 45363, Indonesia; susandy@gmail.com; 3 Faculty of Forestry and Wood Sciences, Czech University of Life Sciences Prague, Kamycka 129, 16500 Praha-Suchdol, Czech Republic; 4 Department of Food Technology, Widya Mandala Surabaya Catholic University, Jl. Dinoyo 42-44, Surabaya 60265, Indonesia; radix@ukwms.ac.id; 5 Faculty of Medicine, School of Nutrition, Universitas Brawijaya, Malang 65145, Indonesia; nia_wirawan.fk@ub.ac.id

**Keywords:** ready-to-use supplementary food, undernutrition, children under five, local food resources, moderate acute malnutrition

## Abstract

Undernourishment is a threat to human health. The prevalence of undernourishment remains alarming, especially among children under five years old in many countries, including Indonesia. Nowadays, the handling of undernourishment has shifted to treatment outside the hospital, utilizing local nutrient-rich foods. At the national level, the utilization of local food resources is a part of the promotion of dietary diversification and the bioeconomy. Ready-to-use supplementary food (RUSF) refers to supplementary foods aimed at improving the nutrition of moderate acute malnutrition (MAM) children under five years old. RUSF biscuit recipes were made using local food resources available in Banten province, Indonesia. To optimize the nutritional profile of the developed RUSF, taro/talas banten were mixed with ground-nut/peanut (*Arachis hypogaea* L.) and mungbean (*Vigna radiata*) as protein and lipid sources and red rice (*Oryza longistaminata*) and maize (*Zea mays*) as carbohydrate sources, and enriched by the local banana Nangka (*Musa textilia*). Two formulations were selected for the pilot testing, namely the taro-peanut and taro-peanut/mungbean RUSF biscuits, made from taro Banten, cereal, peanut and/or mungbean, and local banana. The RUSF biscuit showed promising results, presenting a high level of acceptance and a macronutrient composition that meets the standards for MAM children. However, the RUSF biscuits should be fortified with micronutrient premix to fulfill the dietary requirement for the MAM children. The results of this study provide further development opportunities.

## 1. Introduction

Indonesia has numerous local food resources, including several tubers, cereals, beans, fruits, and vegetables. Due to the lack of information on their potential uses, nutrient content, and the stigma of being inferior foods, some of these local food resources are underutilized. Today, many underutilized foods are gaining popularity because they have nutritionally rich compounds, which can be used to combat malnutrition and food and nutrition insecurity in the country [1,2]. At the same time, Indonesia also experiences a high prevalence of undernutrition. Based on a report by the Basic Health Survey [3,4], the proportion of stunting, indicated by the height-for-age Z-score (HAZ) less than or equal to −2 standard deviation (SD), among children under five years of age in Indonesia was in the range of 30.8–36.8%, which was higher than all of Southeast Asia (25.7%) and even the global prevalence (22.2%) [5]. Meanwhile, the proportion of wasting (weight-for-height Z-score (WHZ) less than or equal to −2 SD) was about 10.2–13.6%, whereas the proportion among underweight children (weight-for-age Z-score (WAZ) less than or equal to −2 SD) was 17.7–18.4%.

Strategies to manage all forms of malnutrition, including severe acute malnutrition, should be food based. Sufficient amounts of energy and various macro and micronutrients, are essential for the human body’s proper growth and development [6,7]. The reduction in the proportion of severe malnutrition (indicated as severely wasted), particularly in children, over a specified period, should be the first indicator of the success of strategies to manage malnutrition [8]. The concept of ready-to-use foods is involved in the prevention and rehabilitation of undernourished children in the community. Ready-to-use food (RUF) is defined as any food designed to be directly consumed from the packet without the need for cooking, dilution, or other preparation. RUF is, therefore, an umbrella term that includes ready-to-use therapeutic food (RUTF) and ready-to-use supplementary food (RUSF). RUTF and RUSF consist of energy-dense, micronutrient-enhanced pastes used in therapeutic feeding that contain all or a portion part of the energy and nutrients necessary for rapid catch-up growth of those with severe acute malnutrition [9,10]. Peanut-based RUTF has been proven successful in treating severe acute malnutrition. Today, peanut-based RUTF is increasingly used to prevent young child malnutrition [8]. The provision of RUTF and RUSF for the treatment of severe acute malnutrition has been studied in various countries, e.g., in Ethiopia [7], Indonesia [11], Nigeria [12], Vietnam [13], and Bangladesh [14]. In addition, the provision of RUSF in a feeding program among mild and moderately wasted children has been reported in Indonesia [15,16]. Those RUTF and RUF were comparable to the international standard [17,18].

More recently, local food resources have been promoted for ready-to-use supplementary food (RUSF) production, including the issues of food security, sustainability, and sovereignty to access foods [15,19]. Meanwhile, the Indonesian government has also encouraged the utilization of diverse local foods and staple foods [20] to support the development of underutilized local foods.

Among many commodities, taro (*Xanthosoma undipes* K. Koch) (local name: Talas banten) can potentially be used as an RUSF ingredient due to its high energy, vitamin, and mineral contents. Talas banten can be easily found as a wild plant or cultivated by small-scale farmers in the Pandeglang District, Banten province, Indonesia. Due to its unpopular nature, limited publications can be found regarding its cultivation, post-harvest, utilization, and economical aspects. Studies have shown that talas banten is a rich source of starch, which can substitute for rice as a staple food [21]. Meanwhile, research has also analyzed the potential economic contribution of talas banten in relation to its rich nutritional profile [22].

The study aimed to develop a ready-to-use supplementary food (RUSF) from the composite flour of local food resources in Indonesia following the guidelines for the management of moderate acute malnourished children under five years of age. Limited reports have revealed the use of talas banten as a food ingredient [23,24]. Thus, to optimize the nutritional profile of the developed RUSF, talas banten were mixed with groundnut/peanut (*Arachis hypogaea* L.) and mungbean (*Vigna radiata*) as protein and lipid sources, red rice (*Oryza longistaminata)* and maize (*Zea mays*) as carbohydrate sources, and banana (*Nangka*) (*Musa textilia*) as a mineral source.

## 2. Materials and Methods

The biscuit was selected as the food carrier of the newly developed RUSF from the composite flour of the local foods because the Indonesian children favored different types of ready-to-eat snacks, including biscuits [25,26]. In addition, research investigating the effectiveness of locally produced RUSF biscuits distributed among mildly and moderately wasted children on Nias island, Indonesia, reported promising results in terms of acceptance [15,16].

### 2.1. Analysis of Raw Materials (Local Food Resources)

#### Source of Raw Material and Preparation

The local food resources used in the study were taro (*Xanthosoma undipes* K. Koch), red rice (*Oryza sativa*), peanut (*Arachis hypogaea*), mungbean (*Vigna radiata*), banana (nangka) (*Musa textilia*), and maize (*Zea mays*). They were cultivated and made into flour by a local farmer in Pandegelang District, Banten province, Indonesia. Other consumable materials, such as salt, egg yolk, cooking oil, and refined sugar, were purchased from the supermarket.

For the local food resources in the form of tuber (taro) and banana, the flour production was based on the common practice of the local farmers. The method involved washing, peeling, cutting, drying, crushing, grinding, and sieving. The local food resources in the form of cereals (red rice, maize) and beans followed sorting, drying, and the same procedure as described above. All local food resources processed into flour were first sun-dried until they achieved about 12–15% of the remaining moisture content.

### 2.2. Product Development and Evaluation

The formulation of RUSF biscuit recipe involved the combination of several food resources, as described above, in addition to other ingredients, such as milk powder, wheat flour, sugar, salt, and whole egg. Nutrisurvey version 2007, Nutrisurvey, Willstätt-Legelshurst, Baden-Württemberg, Germany was used with the Indonesian source food database for the ingredient composition in the proposed RUSF biscuit recipes to meet the WHO standard and other recommended formulations. The RUSF biscuits were prepared and baked in the Department of Nutritional Science’s laboratory and the Department of Applied Nutrition Science/Dietetic’s metabolic kitchen at the University of Hohenheim, Stuttgart Germany.

The dry ingredients were first mixed in each recipe until a homogeneous mixture was formed. Then, the liquid ingredients were added, and the dough was formed. Vitamin and mineral mix powder (Table 1) produced by Pfizer (Berlin, Germany) (Centrum^TM^) was added to the dough to complete the preparation of the RUSF biscuits. The dough was molded into spheres and then baked in an electric oven (Siemens, Munich, Bayern, Germany) at a temperature of 150 °C for 15 min [16]. After cooling down to room temperature, the biscuits were placed in airtight containers and kept at minus 80 °C until further analysis could be performed. The process of making RUSF biscuits included the preparation of ingredients, weighing, mixing (manual), molding (7 to 8 g per biscuit per 100 g), baking (15 min, 150 °C), and packaging.

### 2.3. Determination of the Nutrient Composition

The chemical properties of the flour (raw materials) and RUSF biscuits without vitamin and mineral mix, as well as micronutrient stability test ofthe fortified RUSF biscuits before and after baking, were analyzed in duplicate at the Core Facility, University of Hohenheim, an accredited testing institute (DIN EN ISO/IEC 17025:2005), with state-of-the-art equipment coupled with well established laboratory protocols. The composition was determined using the Commission Regulation (European Union/EU) [27] for protein, fat, ash, and moisture content. The carbohydrate content was calculated using the difference [28]. The energy value was estimated using a formula of (protein × 4 + carbohydrate × 4 + fat × 9) and presented in kcal.

Vitamins (vitamins A, B1, B2, E) were analyzed in triplicate at the Institute of Nutritional Science University of Hohenheim using the high-performance liquid chromatography (HPLC) method, Shimadzu (Shimadzu Corporation, Kyoto, Japan), with the methods, developed and optimized by Wald et al. [29], Triller [30], and Irías-Mata et al. [31], respectively. All the reagents used were purchased from either Sigma-Aldrich Chemie (Taufkirchen, Germany) or Merck (Darmstadt, Germany) unless stated otherwise. Vitamin A was calculated based on retinol equivalent (RE) conversion factors [32], where:

1 RE = 1 µg retinol

   = 6 µg beta-carotene

   = 12 µg other carotenoids

Minerals (Ca, Mg, K, Na, P, Fe, Zn, Cu, Mn, I, Se) were analyzed using the Commission Regulation (EU) (Europian Commision, 2009) using the atom-emission spectrometers VistaPRO ICP-OES (Varian Inc., Palo Alto, CA, USA) for Ca, Mg, K, Na, P; and ICP-MS NexION 300X (PerkinElmer, Inc., Boston, MA, USA) for Zn, Se, Fe, Cu, and iodine. The results of the proximate analysis of the newly developed RUSF biscuits were compared to the proposed recommended nutrient intake for well nourished Indonesian children aged 1 to 3 and 4 to 6 years old [33] and tolerable upper limit of nutrients for moderate acute malnutrition (MAM) children for foods (in 1000 kcal) [17] (Table 2).

### 2.4. Determination of Physical Properties

The diameter and thickness of the RUSF biscuits were determined using a digital Vernier caliper (Mitutoyo Co., Kawasaki, Japan) with the accuracy of ±0.01 mm. An average of five values for each of three replications was taken for each sample set and reported in millimeters. The spread ratio was calculated by dividing the diameter by the thickness.

Color analysis of biscuit was carried out using a Chroma meter Minolta CR-400 (Konica Minolta Sensing Americas, Inc., Ramsey, NJ, USA) based on the CIA L*, a*, and b* color system. L* measures black to white (0–100), the a* value measures redness when positive, and the b* value measures yellowness when positive.

### 2.5. Sensory Evaluation

Sensory evaluation of the samples was conducted with 66 untrained, purposively chosen panelists composed of students and staff of the Institute of Nutritional Science, University of Hohenheim, and the Indonesian community in Stuttgart, Germany. Five coded RUSF biscuits were presented to each panelist at each session. The panelists assessed the RUSF biscuits for appearance, aroma, color, texture, mouth-feel, aftertaste, and overall acceptability using a seven-point hedonic scale, with 1 and 7 representing the lowest score (dislike very much) and the highest score (like very much), respectively [34].

### 2.6. Data Analysis

Food data were converted into per-100 g of dry weight based on the moisture content of each sample. Data on the nutritional composition of the local food resources and RUSF biscuits were analyzed using the SAS statistics version 9.4 (SAS Inst. Inc., Cary, NC, USA). The selected ‘nutrients’ mean differences in each recipe were compared and analyzed using the independent *t*-test. The means of the RUSF biscuit recipes were compared with the international recommendations (Table 1) using an independent *t*-test. A *p*-value of less than 0.05 was used to designate the statistical significance in all analyses.

## 3. Results

### 3.1. Nutritional Composition of Local Food Resources

Various food sources resulted in different nutritional compositions, which complemented each other when combined to produce better products with better nutritional content. The nutritional information on the local food resources used as the ingredients in the newly developed RUSF biscuits is shown in Table 3. Among the ingredients in the RUSF biscuit recipes, the mungbean flour had the highest protein and total mineral content, while the peanut had the highest fat and energy content. For the mineral content, mungbean had the highest calcium, phosphor, and magnesium, while banana (ambon) had the highest iron content and taro flour had the highest zinc values. With respect to vitamins, maize had the highest pro-vitamin A lutein and zeaxanthin content, while taro had high α- and β-carotene, as well as vitamin B (thiamine and riboflavin).

Originally, 12 RUSF biscuit recipes were developed. After preference evaluation at the Institute of Nutritional Sciences, University of Hohenheim, Stuttgart Germany, confirmed by the field test in Indonesia, the two best RUSF biscuits were selected. The two selected recipes contained similar ingredients. To distinguish the differences, the selected cereal/tuber nut/bean-based RUSF biscuit formulations were named taro–peanut and taro–peanut/mungbean-based, according to the most distinctive ingredients in each recipe (Table 4).

### 3.2. Nutritional Composition of the Biscuit Recipes

The nutrient compositions of the taro–peanut and taro–mungbean-based biscuits are shown in Table 5. No statistically significant difference was found in most of the nutrients (in energy, protein, vitamin A, E, thiamine, riboflavin, magnesium, and zinc). The phosphor and iron composition in the taro–peanut/mungbean-based RUSF formulations were significantly higher than the taro–peanut-based formulation in contrast to calcium content.

**Figure 1:**
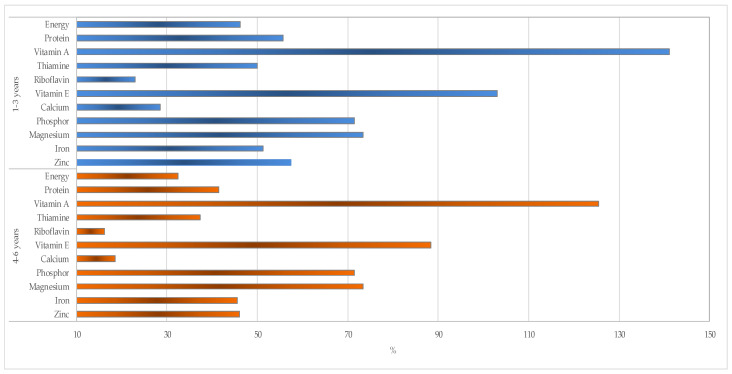
Percent fulfillment of the selected nutrients of taro–peanut/mungbean-based RUSF biscuit based on the well nourished Indonesian children [33].

**Figure 2:**
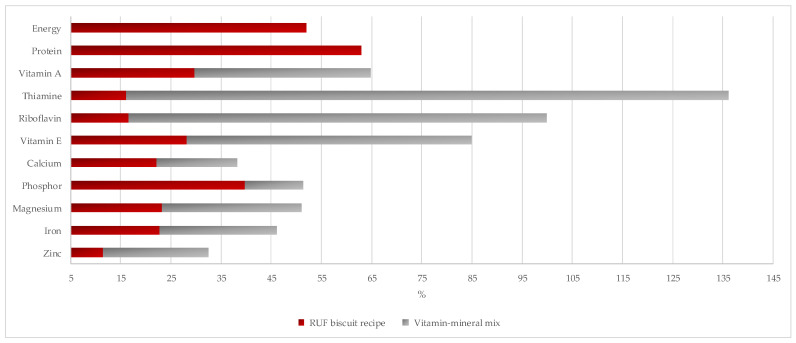
Percent fulfillment of selected minerals of taro–peanut/mungbean-based original recipe and after fortification by the vitamin–mineral mix based on the recommended intake for MAM children [17].

To understand the fulfillment of the two selected recipes according to the dietary recommendations for well nourished [33] and MAM children [17] in Indonesia, the taro–peanut/mungbean-based formulation was selected for further analysis. The majority of the selected nutrients of the taro–peanut/mungbean-based formulation were still below 70% of the recommendation for the well nourished Indonesian children aged between one and five years old, except for vitamin A, vitamin E, phosphor, and magnesium (Figure 1). The gap between the selected nutrients in the taro–peanut/mungbean-based recipe compared to the international recommendation for MAM children was escalated because of the increased dietary requirement targeting catch-up growth. Hence, the vitamin and mineral mix was added to form the RUSF biscuits (Figure 2).

**Figure A1:**
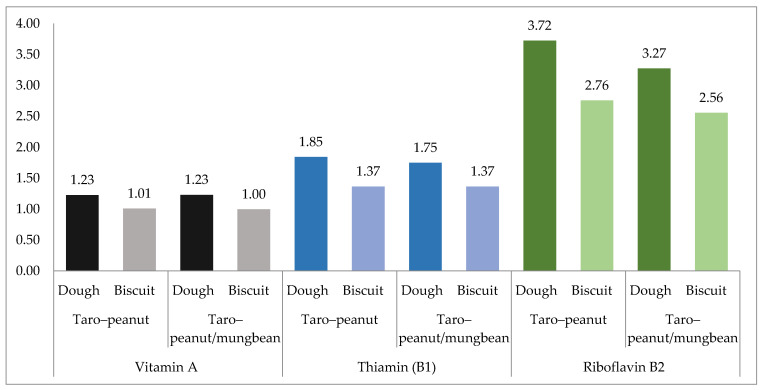
Stability Test of Selected Vitamins in RUSF Biscuit Fortified Micronutrient Premix.

The vitamin losses of RUSF biscuits during the baking process were approximately between 15 and 28% (Figure A1). Vitamin A loss varied between 15 and 22%, while Vitamin B (thiamin and riboflavin) ranged between 23 and 28%. Furthermore, Vitamin E content in the RUSF biscuits was reduced between 23 and 26% after baking. The mineral content of the RUSF biscuits was relatively stable before and after baking.

### 3.3. Physical Properties and Sensory Evaluation of RUSF Biscuit Recipes

The dimensions (diameter, thickness, and spread ratio) and color (L*, a*, and b*) of the RUSF biscuits are shown in Table 6. The mean diameter of the RUSF biscuits was 40–41 mm, with a 7 mm thickness and a spread ratio of 6. The dimensions d not differ significantly because all the RUSF biscuits were formed using one mold. Further, since the amount of wheat was the same in all recipes, the expansion of the biscuits was quite identical. The diameter, thickness, and spread ratio were not significantly different.

The positive values of a* and b* indicate the predominance of redness and yellowness in the RUSF biscuit. The biscuits’ color changed to dark brown (final color) from creamy yellow as in the dough. The brightness value (L*) was above 50 (L* = 0 is black and L* = 100 is white) for all the recipes.

Panelists of the sensory evaluation consisted of 34 females and 32 males, aged from eight to 50 years with an average of 30 years, living in Germany. The comparison of the preference scores by the panelist to all the parameters presented the RUSF biscuits’ overall choices from the composite flour of local food resources (Figure 2). There were no significant differences in the attributes of appearance, aroma, color, texture, mouth-feel, and aftertaste. All the parameters showed a slight difference in the consumers’ acceptance. All the RUSF biscuits’ sensory attributes obtained similar scores within the range of 4 to 5 (neutral to like moderately). Almost all indicator scores in the sensory evaluation of the taro–peanut-based exceeded those of the taro–peanut/mungbean-based RUSF recipes, but the overall score of taro–peanut (4.6) was slightly higher than taro–peanut/mungbean-based RUSF biscuits (4.5).

## 4. Discussion

Malnutrition by means of undernutrition primarily affects children under the age of five because of their more demanding dietary requirements. Undernourishment in childhood could affect children’s overall cognitive development, school performance, lifetime earnings, and vulnerability to infectious and chronic diseases in adulthood (higher risk of death and illness) [35]. The provision of RUSF in a community-based feeding center is essential in preventing and rehabilitating MAM children. RUSF can be made into multiple food types [36]. RUSF biscuits are easy to prepare and favored by children under five years of age. The utilization of local foods as the source of ingredients follows the national and provincial government strategy to promote diversification in staple foods and the utilization of underutilized local foods [20]. The composite flour mix of the local foods with wheat flour and milk powder resulted in biscuits high in vitamins A and E, as well as a moderate content of phosphor and magnesium for children under the age of five. In addition, the cereal/tuber nut/bean-based formulation depicted the presence of other micronutrients (e.g., thiamine, riboflavin, calcium, iron, and zinc), although at a low level (Figure 2 and Table 4). The locally-made RUSF biscuits can be used to replace the energy-dense snacks that are usually consumed by Indonesian children [25,37]. Based on the selection criteria weighting scores (nutritional composition of all RUSF biscuits, variety of local food resources used in the formulation, and acceptance of sensory testing) applied in the study, of 12 RUSF biscuits, five recipes were selected for further research in the field. After the pilot testing in Banten, two recipes, namely taro-peanut and taro-peanut/mungbean-based, were chosen by the local women [38].

**Figure 3:**
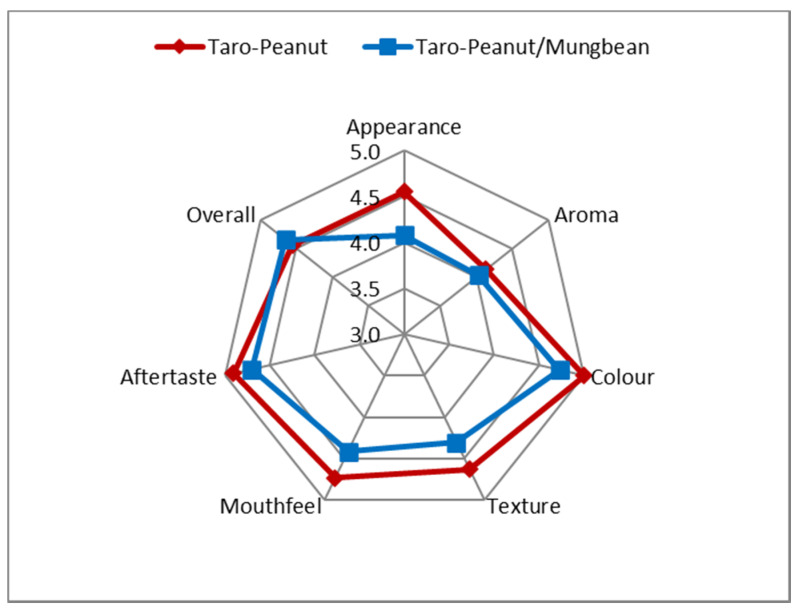
Spiderweb of sensory evaluation scoring of two selected RUSF biscuit recipes.

Comparison of the nutritional composition of the recipes with the international standard for prevention and treatment of MAM children showed that the selected nutrients were below the recommenede levels. Therefore, to enhance the nutrient content of the RUSF bisquit, a vitamin and mineral mix was added (Figure 3), as also reported in the development of the local RUSF/RUTF in Ghana, Ethiopia, Pakistan, India [39], Senegal [40], Nigeria [12], Cambodia [41], and Nias [15]. The provision of RUSF biscuits on Nias among moderately wasted children was prescribed as snacks and did not replace home-based meals. Therefore, nutrition education to improve the children’s diet was also carried out among the caregivers on Nias [15]. Similarly, the intention of developing the cereal/tuber nut/bean-based RUSF biscuits was not to replace the habitual diet and home-based meals of the children. The nutrients that exceeded 70% of the dietary recommendation for MAM children were thiamine, riboflavin, and vitamin E, and the thiamine content was above 100% of the standard. In contrast, all selected minerals in Figure 3 were below 70% of the dietary recommendation for MAM children. To improve the mineral composition of the biscuits, it is recommended to add local foods rich in the missing minerals to the recipes, e.g., chicken’s liver, dried fish, etc. The children’s home-based meals are expected to cover the nutrient gap.

Thiamine and riboflavin are water-soluble and heat-sensitive. Thus, there may be some vitamin losses after baking. The loss of thiamine, one of the most unstable B vitamins, was about 15–20% after baking. Riboflavin, which is more stable during thermal processing than thiamine, was reported to experience around 5–10% loss during baking [42]. Thiamine is stored in the body in a very limited amount, yet the nutrients have an important metabolic role as a cofactor of many enzymes. Thiamine deficiency is more profound among polished rice consumers [43,44,45]. Furthermore, within populations lacking the consumption of dairy products, such as Indonesian populations, riboflavin deficiency is predicted to be prevalent [46]. To prevent the loses of vitamins during processing, the high amount of thiamine and riboflavin were added. More detail vitamin loses of the RUSF biscuits after baking are presented in Figure A1.

Concerning the physical properties, the biscuit spread ratio had a smaller score than biscuits made from cassava/soybean/mango composite flour, as shown by the authors of [47]. The spread ratio was also smaller than biscuits made with the composite flour of germinated pigeon pea, fermented sorghum, and cocoyam flours, which was reported by the authors of [48] as 14 to 20 percent. The spread ratio of RUSF biscuits from the composite flour of local food resources is quite similar to the biscuits from [49], which is likely due to some identical ingredients in the biscuit recipe, particularly banana and taro. The spread ratio is an important parameter for biscuits. A higher spread ratio is preferable by consumers. Several factors are responsible for higher spread ratios, such as hydration properties, protein content, water binding ability, lipid and fiber content, the presence of non-starch polysaccharides, and the extensibility property of flour [50].

While the newly developed recipes still included other non-local food resources, e.g., wheat flour and milk powder, the foods are available in small shops across the country, even in a small sachet. The wheat flour has gluten, which can trap the dough’s oxygen and expand the biscuit spread after the baking process, causing the crunchy but soft texture of the biscuits. Meanwhile, milk powder provided a tasty flavor and improved the nutritional value of the biscuits. In Indonesia, especially in remote areas, milk powder is costly and has a high risk of bacterial contamination. Alternative RUSF formulations with small amounts of milk powder can be produced by the community, small-scale industries, or organizations. Furthermore, low-income families with a high risk of malnutrition could afford to make this formulation at the household level with minimum support. The previous study on mildly to moderately wasted children on Nias, Indonesia, reported the effectiveness of locally produced RUSF biscuit using the locally available food resources and limited technology intervention. These results were confirmed by the weight-for-height Z-score (WHZ) with standard deviations (SD) from >−2 to <−1.5 [15]. The RUSF’s local production would help reduce the associated supply challenges, costs, and lead times on delivery, and would stimulate local economies by transferring skills and local agricultural products, thereby increasing the beneficial social impacts and sustainability [51]. Moreover, the use of locally available food resources would increase the sustainability of the program. It would allow the higher participation of the community and increase the sense of belonging to the program, awareness on the nutrition potential of the locally available food resources. In turn, the use of locally available food resources would increase the consumption of household food production and income, reduce market prices, and create shifts in consumer preferences and in the control of resources [52]. In conjunction with nutrition education, the use of locally available food resourcs would improve the availability, access to, and utilization of foods with a high content and bioavailability throughout the year [53]. Color is one of the parameters used for quality control in the baking process and an important attribute for the biscuits’ acceptability because it can pique the appetite. Several factors have been reported to affect the color’s development on the product’s surface, including the moisture content, temperature, air velocity, and heat transfer [54]. There is no significant difference observed in the lightness, redness, and yellowness of both of the formulated biscuits (Table 4), which might be due to the similarity of factors that contributes to the color development of biscuits, such as protein and reduced sugar content, as well as moisture content, temperature, air velocity, and heat transfer.

There were no significant differences in the attributes of appearance, aroma, color, texture, mouthfeel, and aftertaste. All the parameters showed a slight difference in the consumer’s acceptance. A similar result was shown in a study comparing locally produced RUTF, with the standard peanut-based RUTF containing powdered milk in Ethiopia, Ghana, Pakistan, and India [39]. The acceptance of local RUTF was quite similar to the standard RUTF. As a target group, the young children presented the liking score of about 3.5 to 4.6 (neutral to slightly like). The study of the development of milk-free soybean–maize–sorghum-based RUTF (SMS-RUTF) using locally grown ingredients in Kenya [55] showed that the preferences of children aged 4 to 11 years with the product were between ok and very happy. The development of locally produced fish-based food for undernourished children in Cambodia confirmed the acceptability of the target group, with the results indicating that they liked the product. The study in Malawi which compared locally produced RUTF with imported RUTF, showed that the locally produced RTUF was more liked by the children [56]. The short-range of the scores resulting from the panelist preferences in our study indicated that all recipes were acceptable (Figure 2).

In addition, food processing that includes baking may interfere with reducing nutrient content, as heat processing, particularly by boiling, may lead to essential changes in the food’s weight and nutrient content. The average retention factor of milk-based food during heating (i.e., frying using an oven) for vitamin B1 and B2 is 0.75 and 0.9, respectively. For egg-based food, the retention factor is 0.95 for vitamin B1 and B2. For legume-based dishes, the retention factor is 0.65 to 0.8 for vitamin B1 and 0.8 to 1.0 for vitamin B2. However, retinol/carotenoid and vitamin E remain relatively unchanged during the heating process, with a retention factor of 1.0 for all types of food [57].

The selected fat- and water-soluble vitamins losses were examined after the baking process, as depicted in Figure A1. The vitamin A loss (15 to 22%) in the fortified RUSF biscuits was comparable to the reduction of vitamin A during storage (15 to 34%) [58] and cooking (25%) [59]. The water soluble vitamin B1 and B2 content were reduced approximately around 23 to 28%. The results were lower than the vitamin B1 loss (50%) after extrusion in 80 °C and the reduction of vitamin B2 in foods after undergoing palette and extrusion process (35%) [60]. The removal of vitamin E in the RUSF biscuits (23 to 26%) was also lower compared to the vitamin E loss in foods during storage (32%) [58]. Besides the short baking process (15 min), the micronutrient premix as the vitamin source in the fortified RUSF biscuits has higher stability than the original source of micronutrient in foods. To balance the vitamin loss during the baking process, a higher concentration of micronutrient premix was added to ensure the RUSF biscuits were in accordance with the nutrient requirements for the MAM children.

The cost of RUSF biscuits from local food resources in Banten was calculated according to the local price for the ingredients. The cost of RUSF biscuits in Banten was higher (0.56 Euro per 100 g) than the RUSF biscuits on Nias island (0.15 to 0.16 Euro per 100 g [61]. The micronutrient premix on Nias was donated by the DSM. Therefore, for the biscuit price estimation, the cost of the micronutrient premix included only the custom fee and transportation expenses from the airport in Jakarta (the capital city of Indonesia) to Nias Island, while the actual price of the premix itself was not included in the calculation [62].

The RUSF biscuit price of 0.56 euro per 100 g was higher than the commercial biscuits in Indonesia (around 0.35 to 0.45 euro per 100 g). Of the total biscuit price, approximately 70% of the cost was for the micronutrient premix. The slightly higher cost was compensated by having the micronutrients content needed for the growth of the undernourished children. Hence, the promotion of the RUSF biscuits production and utilization in the country should be accompanied by nutrition education. The importance of the nutrients fortified in RUSF biscuits for the under-fives should be explained so that the caregivers can select nutritious snacks for their children. To combat undernourishment among children in Indonesia, support from the government and other stakeholders is still needed to ensure the program’s sustainability, especially for the micronutrient premix supply.

In the study on Nias, the RUSF biscuits from local food resources resulted in a low proportion (6 to 7%) of the food supplement cost spent for the entire intervention programs [62]. While some of the ingredients on Nias were the same as in Banten, the underutilized indigenous foods (e.g., taro Banten and local banana) rich in micronutrients were used in the current RUSF biscuits. As the RUSF biscuit production utilizing the local food resources can potentially promote the bioeconomy in the region thus, a cost-benefit calculation from economic, social, and environmental point-of-view is recommended to analyze the prospective production at a larger scale. For instance, the cost and benefit of establishing a home industry of RUSF biscuits, cultivating the foods in a barren land for providing the ingredients, using environmentally friendly packaging to reduce plastic pollution, etc.

## 5. Conclusions

The newly developed biscuit recipes showed promising results as a nutrient-dense snack for well nourished children. However, the results showed that the two presented recipes of RUSF biscuits are still below the international recommendation for the prevention and rehabilitation of MAM children. Hence, they need to be fortified with vitamins and minerals. Our study indicated that all RUSF biscuit recipes made from the local resources were acceptable in terms of physical properties, color, taste, and dimension. The consumption of RUSF biscuits should not replace the consumption of home-based meals among children. Instead, the biscuits should be given as nutritious snacks made from local ingredients. Therefore, the provision of RUSF biscuits for the prevention and rehabilitation of MAM children should be accompanied by health/nutrition education to the caregivers to provide a well balanced diet for the children.

## Figures and Tables

**Table 1:** Vitamin and mineral mix used in the study *.

Micronutrient	Unit	Premix
Selected micronutrients analyzed in the study		
Vitamin A	µg	666.7
Vitamin B1 (Thiamine)	mg	1.2
Vitamin B2 (Riboflavin)	mg	1.5
Vitamin E	mg	12.5
Calcium	mg	135.0
Phosphorus	mg	104.2
Magnesium	mg	83.3
Iron	mg	4.2
Zinc	mg	4.2
Other micronutrients		
Vitamin D	µg	8.3
Vitamin C	mg	83.3
Vitamin B6	mg	1.7
Vitamin B12	µg	2.1
Niacin (B3)	mg	16.7
Biotin (B7/B8)	µg	52.1
Folic Acid	µg	166.7
Vitamin K	µg	25.0
Phantotenate (B5)	mg	6.3
Iodine	µg	83.3
Copper	mg	0.4
Selenium	µg	25.0

* 2 g premix was added in 100 g of RUFS biscuit dough.

**Table 2:** Selected micronutrient recommendations for well nourished and MAM children.

Micronutrient	Unit	Recommended Nutrient Intake		
		Well Nourished Children [33]		MAM Children [17]
		1 to 3 years	4 to 6 years	
Macronutrients				
Energy	Kcal	1125	1600	1000
Protein	G	26	35	35
Vitamins				
Vitamin A	µg	400	450	1900
Vitamin B1 (Thiamine)	µg	0.6	0.8	1
Vitamin B2 (Riboflavin)	µg	0.7	1	1.8
Vitamin E	Mg	6	7	22
Minerals				
Calcium	Mg	650	1000	840
Phosphorus	Mg	500	500	900
Magnesium	Mg	60	95	300
Iron	Mg	8	9	18
Zinc	Mg	4	5	20

**Table 3:** Selected nutrients of the local food resources from Banten province, Indonesia, per 100 g ^1^.

Local Food Resources	Taro *(Xanthosoma undipes K. Koch)*	Red Rice (*Oryza sativa)*	Maize (*Zea mays*)	Peanut (*Arachis hypogaea*)	Mungbean (*Vigna radiata*)	Banana (*Nangka*) (*Musa textilia*)
Macronutrients						
Energy (kcal)	353.9	361.6	351.7	622.3	355.8	320.9
Protein (g)	11.7	10.6	10.6	30.7	26.4	3.6
Fat (g)	1.5	2.9	3.9	50.9	1.3	0.5
Carbohydrate (g)	73.5	73.1	68.5	10.3	59.6	75.5
Vitamins						
Lutein (µg)	411.7	16.7	1279.3	29.3	527.9	ND
Zeaxantine (µg)	5.2	ND	2320.5	6.5	20.2	ND
Alfa Carotene (µg)	1247.7	44.1	60.1	53.3	69.8	10.1
Beta carotene (µg)	1762.4	169.1	575.3	177.6	273.9	162.7
Retinol Equivalent (µg)	397.7	31.85	100.9	34.04	51.46	27.97
Thiamine (µg)	0.5	1.5	3.3	2.4	1.5	0.5
Riboflavin (µg)	69.6	31.9	186.4	59.2	121	111.4
Mineral						
Ca (mg)	43	11	6.8	92	132	15.1
P (mg)	84	337	353.3	376	367	53.8
Mg (mg)	33	112	157.7	168	189	68.3
Fe (mg)	7.4	2	3	4.6	6.7	2.2
Zn (mg)	3.8	2.5	2.4	3.3	2.7	0.4

^1^ ND = not detected, retinol equivalent calculation: (µg α-carotene/12) + (µg β-carotene/6) [32].

**Table 4:** Recipes of cereal/tuber nut/bean-based biscuits from local food resources, per 100 g.

Ingredients (in % of Weight)	Taro–Peanut	Taro–Peanut/Mungbean
Wheat Flour	6	6
Talas Powder (Taro)	6	8
Red Rice	5	5
Peanut/groundnut (without skin/peeled)	14	16
Sugar powder	18	18
Whole milk powder	14	12
Chicken egg yolk	19	19
Palm oil	7	7
Salt	1	1
Mungbean powder		4
Banana powder (Nangka)	5	
Maize powder	5	4
Total	100	100

**Table 5:** Proximate composition of the cereal/tuber nut/bean-based biscuit recipes from the composite flour of local food resources, per 100 g ^1^.

Nutrients ^2^	Taro–Peanut	Taro–Peanut/Mungbean	*p*-Value
Macronutrient			
Energy, kcal	529.7 ± 0.4	533.0 ± 0.1	0.993
Protein, g	14.4 ± 0.00	14.5 ± 0.10	0.152
Vitamin			
Vitamin A, µg	565.5 ± 7.2	564.7 ± 5.3	0.850
Thiamine, mg	0.17 ± 0.05	0.17 ± 0.17	1.000
Riboflavin, mg	0.31 ± 0.00	0.30 ± 0.01	0.145
Vitamin E, µg	6.2 ± 0.4	6.2 ± 0.3	0.964
Mineral			
Calcium, mg	187.3 ± 0.9	186.0 ± 0.1	0.433
Phosphor, mg	354.7 ± 0.3	357.6 ± 0.1	0.590
Magnesium, mg	69.8 ± 0.5	69.8 ± 0.2	0.799
Iron, mg	3.6 ± 0.2	4.1 ± 0.0	0.002
Zinc, mg	2.4 ± 0.2	2.3 ± 0.1	0.270

^1^ Values are in the mean ± standard deviation of the triplicate determination from each RUSF biscuit sample. Data were analyzed using the independent *t*-test to determine the statistically significant differences (*p* < 0.05). ^2^ Values of macronutrients are on a dry basis. kcal = kilocalorie. mg = milligram, µg = microgram.

**Table 6:** Physical properties of RUSF biscuits from local food resources ^1^.

RUSF	Diameter (mm)	Thickness (mm)	Spread Ratio	Color		
				L*	a*	b*
Taro–peanut	40.4 ± 0.6	7.1 ± 0.5	5.7 ± 0.4	58.5 ± 2.1	8.7 ± 0.8	26.8 ± 0.7
Taro–peanut/mungbean	40.6 ± 0.7	7.1 ± 0.6	5.8 ± 0.5	55.3 ± 0.7	11.5 ± 0.3	24.1 ± 0.8
*p*-value	0.670	0.772	0.997	0.003	0.009	0.699

^1^ Values are mean score ± standard deviation of an average of three replications for each set of the recipe. Mean differences were compared and analyzed using the independent *t*-test. *p* < 0.05 = statistical significant different.
